# Counties not countries: Variation in host specificity among populations of an aphid parasitoid

**DOI:** 10.1111/eva.12759

**Published:** 2019-01-31

**Authors:** Keith R. Hopper, Sara J. Oppenheim, Kristen L. Kuhn, Kathryn Lanier, Kim A. Hoelmer, George E. Heimpel, William G. Meikle, Robert J. O’Neil, David G. Voegtlin, Kongming Wu, James B. Woolley, John M. Heraty

**Affiliations:** ^1^ Beneficial Insect Introductions Research Unit USDA‐ARS Newark Delaware; ^2^ American Museum of Natural History New York New York; ^3^ Department of Entomology University of Minnesota St. Paul Minnesota; ^4^ European Biological Control Laboratory USDA‐ARS St. Gely du Fesc CEDEX France; ^5^ Department of Entomology Purdue University West Lafayette Indiana; ^6^ Illinois Natural History Survey Champaign Illinois; ^7^ Institute of Plant Protection Chinese Academy of Agricultural Sciences Beijing China; ^8^ Department of Entomology Texas A&M University College Station Texas; ^9^ Department of Entomology University of California Riverside California

**Keywords:** aphid, clinal, evolution, *F*_ST_, genetic differentiation, local adaptation, mosaic, parasitoid

## Abstract

Parasitic wasps are among the most species‐rich groups on Earth. A major cause of this diversity may be local adaptation to host species. However, little is known about variation in host specificity among populations within parasitoid species. Not only is such knowledge important for understanding host‐driven speciation, but because parasitoids often control pest insects and narrow host ranges are critical for the safety of biological control introductions, understanding variation in specificity and how it arises are crucial applications in evolutionary biology. Here, we report experiments on variation in host specificity among 16 populations of an aphid parasitoid, *Aphelinus certus*. We addressed several questions about local adaptation: Do parasitoid populations differ in host ranges or in levels of parasitism of aphid species within their host range? Are differences in parasitism among parasitoid populations related to geographical distance, suggesting clinal variation in abundances of aphid species? Or do nearby parasitoid populations differ in host use, as would be expected if differences in aphid abundances, and thus selection, were mosaic? Are differences in parasitism among parasitoid populations related to genetic distances among them? To answer these questions, we measured parasitism of a taxonomically diverse group of aphid species in laboratory experiments. Host range was the same for all the parasitoid populations, but levels of parasitism varied among aphid species, suggesting adaptation to locally abundant aphids. Differences in host specificity did not correlate with geographical distances among parasitoid populations, suggesting that local adaption is mosaic rather than clinal, with a spatial scale of less than 50 kilometers. We sequenced and assembled the genome of *A. certus*, made reduced‐representation libraries for each population, analyzed for single nucleotide polymorphisms, and used these polymorphisms to estimate genetic differentiation among populations. Differences in host specificity correlated with genetic distances among the parasitoid populations.

## 1 INTRODUCTION

Parasitic wasps are among the most species‐rich groups on Earth, comprising ~600,000 species (Heraty, 2009), and a major cause of this diversity may be local adaptation to host species. Local adaptation can be a major driver of evolution and speciation (Schluter, 2009), and the spatial scale and patchiness of such adaptations reflect that of the environmental factors to which populations are responding. In *Drosophila melanogaster *Meigen (Diptera: Drosophilidae), variation in adaptation to climate can be measured both at the scale of a single valley, reflecting microclimate differences between two sides of a canyon (Michalak et al., 2001) and over thousands of kilometers, reflecting latitudinal clines (Kolaczkowski, Kern, Holloway, & Begun, 2011). In Edith's checkerspot butterfly, *Euphydryas editha *(Boisduval) (Lepidoptera: Nymphalidae), local adaptation occurs as a mosaic, reflecting the patchy distribution of potential host plants (Singer, Wee, Hawkins, & Butcher, 2008). In the burying beetle, *Silpha carinata* Herbst (Coleoptera: Silphidae), local adaptation is clinal, with body size decreasing continuously with altitude (Baranovska & Knapp, 2018).

Although the causes of the speciosity of hymenopteran parasitoids are not well understood, one important factor is their highly specialized adaptation to narrow ranges of hosts (Forbes, Powell, Stelinski, Smith, & Feder, 2009; Strand & Obrycki, 1996). Host specialization may arise because no one genotype is optimal on all host species (Henry, Roitberg, & Gillespie, 2008), and may reflect adaptation to locally abundant hosts. The narrow host ranges of parasitoids make them attractive candidates for the biological control of pest insects, but biological control introductions cannot be safely performed without a clear understanding of variation in host specificity and how it evolves (Henry, May, Acheampong, Gillespie, & Roitberg, 2010; Hopper, Roush, & Powell, 1993).

Despite the potential importance of local adaptation in driving host specificity, little is known about local patterns of parasitoid host use, and the few studies of local adaptation to hosts on different plants or substrates, or from different geographical locations, have reached contradictory conclusions. In *Aphidius ervi* (Hufbauer, 2001, 2002), *Asobara tabida* (Kraaijeveld & Godfray, 2001), and *Leptopilina boulardi* (Dupas, Carton, & Poirie, 2003), scant evidence was found for local adaptation, but studies with *Leptopilina clavipes *(Pannebakker, Garrido, Zwaan, & van Alphen, 2008) and *Leptopilina heterotoma *(Gibert, Allemand, Henri, & Huey, 2010) did find evidence for local adaptation. However, all of these studies dealt with differences in parasitism of a single host species rather than variation in parasitism among host species. Among the few studies of local adaptation of parasitoids to different host species, Gray, Banuelos, Walker, Cade, and Zuk (2007) found that a parasitoid fly showed geographical variation in attraction to the songs of the locally abundant cricket species that it parasitized, and Saul‐Gershenz et al. (Saul‐Gershenz, Millar, McElfresh, & Williams, 2018) found that a parasitic beetle had adapted to the pheromones and male‐patrol heights of locally abundant bee species that it parasitized.

Here, we report on geographical variation in host use among 15 populations of an aphid parasitoid, *Aphelinus certus* Yasnosh (Hymenoptera: Aphelinidae), from its area of origin in eastern Asia, as well as an invasive population from North America. We use these data to address several questions about local adaptation: Do parasitoid populations differ in host range (i.e., the set of species they will parasitize) or host preference (i.e., parasitism rates of individual species within their host range)? Is interpopulation variation related to geographical distance, reflecting clinal variation in the abundances or quality of aphid species? Or, is interpopulation variation independent of geographical distance, as would be expected if differences in aphid abundances or quality, and thus selection, were mosaic? Are interpopulation differences in parasitism related to genetic differentiation, suggesting local adaptation? If so, is the variation associated with genes having different functions?

To answer these questions, we measured parasitism by each *A. certus* population when exposed to a taxonomically diverse set of seven aphid species. We sequenced reduced‐representation genomic libraries for each population to find single nucleotide polymorphisms (SNPs) for analysis of genetic differences. To provide a framework for determining homology among SNP loci, we sequenced and assembled the genome of *A. certus*. We tested the relationships between differences in parasitism versus geographical and genetic distances among these populations. Lastly, we investigated the functions of genes located near reduced‐representation loci with versus without SNPs*. *While we expected that SNP variation might be in linkage disequilibrium with genes affecting host specificity (especially if response to selection were rapid and recent, leaving footprints in the genome), we did not expect there to be a causal relationship between SNP polymorphisms and differences in host specificity.

## 2 MATERIALS AND METHODS

### 2.1 Study system

The genus *Aphelinus* comprises more than 90 species (Hopper et al., 2012; Noyes 2015; Shirley, Woolley, & Hopper, 2017), all of which are endoparasitoids of aphids and are koinobionts (i.e., the host continues to develop after being parasitized). *Aphelinus* species are small (about 1 mm long) and are weak fliers (Fauvergue & Hopper, 2009), searching for hosts and mates primarily while walking (Fauvergue, Hopper, & Antolin, 1995). *Aphelinus *females prefer 2–4th instar aphids for oviposition, but will oviposit in all stages (Rohne, 2002). At 20**°**C, wasps develop from oviposited egg to adult emergence in about three weeks. During their third instar, *Aphelinus* larvae kill their hosts, but leave the host exoskeleton intact, causing it to harden and turn black in a process called mummification (Christiansen‐Weniger, 1994), and adults emerge about one week after pupation. Several *Aphelinus* species are important in biological control of pest aphids (Hopper, Lanier, Rhoades, Coutinot et al., 2017; Hopper, Lanier, Rhoades, Hoelmer et al., 2017; van den Bosch, Schlinger, Dietrick, & Hall, 1959). *Aphelinus certus* females that are 1–2 days old carry a mean of 14 ± 1 (mean ±95% confidence interval) mature eggs (Hopper & Diers, 2014), but the females can produce more eggs throughout their lives, parasitizing a total of 119 ± 10 aphids during a two‐week median lifetime in the laboratory.

*Aphelinus* *certus* was described from collections during 1935 in Primorskiy Territory in far eastern Siberia (Yasnosh, 1963). Populations of *A. certus* from China, Korea, and Japan are reproductively compatible and form a single clade in a phylogeny of the *A. varipes* complex based on DNA sequence data (Heraty et al. 2007). *Aphelinus certus* appears to have recently colonized North America and spread rapidly to reach high levels of parasitism of the soybean aphid, *Aphis glycines* Matsumura (Hemiptera: Aphididae), an invasive pest with which *A. certus* may have been introduced (Heimpel et al., 2010). This parasitoid was first collected in Connecticut in 2004, that is four years after the soybean aphid itself was detected in North America*. Aphelinus certus *was subsequently found in Ontario, Canada, in 2007 (Frewin et al., 2010), although several surveys of parasitoids of soybean aphid did not detect it during 2003–2006 in Michigan or further west (Noma & Brewer, 2008). Since then, *A. certus* has invaded Minnesota and other midwestern states where soybean is a major crop and soybean aphid is a major pest. Parasitism levels are sometimes very high, leading to suppression of the abundance of soybean aphid (Kaser & Heimpel, 2018).

### 2.2 Insects and host plants

During exploration for parasitoids to introduce against the soybean aphid, we collected *A. certus* (as parasitized aphids) from 15 populations in 12 locations in Asia, and we also collected one invasive population in eastern Pennsylvania (Table S1). The parasitoids were brought to the containment facility at the USDA‐ARS, Beneficial Insect Introductions Research Unit, Newark, Delaware, under permits from the United States Animal and Plant Health Inspection Service, Plant Protection and Quarantine, and cultures were started with the population sizes shown in Table S1. To maintain genetic variation under laboratory rearing (Hopper et al., 1993; Roush & Hopper, 1995), each culture was split after one generation into 4 subcultures, each of which was kept at an adult population size >200. The parasitoids were reared on *A. glycines* on soybean in plant growth chambers (model AR66–2L, Percival Scientific, Perry, IA) at 20°C, 50%–70% relative humidity, and 16:8 hr (L:D) photoperiod. When experiments were conducted, the parasitoids had been in culture 6–44 generations.

We measured parasitism of seven aphid species in five genera and two tribes on four plant species in four plant families. In the tribe Aphidini, we used *Aphis glycines* Matsumura on *Glycine max* (L.) (Pioneer 91Y70; Fabaceae), *Aphis gossypii* (Glover) on *Gossypium hirsutum* L. (SG105; Malvaceae), *Rhopalosiphum maidis* (Fitch), *Rhopalosiphum padi* (L.), and *Schizaphis graminum* (Rondani) on *Hordeum vulgare* L. (Lacey; Poaceae); in the tribe Macrosiphini, we used *Diuraphis noxia* (Kurdjumov) on *H. vulgare* and *Myzus persicae* (Sulzer) on *Raphanus sativus* L. (Cherry Belle; Brassicaceae). These aphids span the phylogeny of aphids reported as hosts of the *A. varipes* complex, of which *A. certus *is a member. Also, these aphid–plant combinations provide contrasts of aphids in the same versus different genera on the same versus different plant species. Six of these aphid species have distributions that overlap the geographical range of the populations we studied (Blackman & Eastop 2006), so these aphids and parasitoids are likely to have been in contact for at least 10,000 years, that is since the end of the last glaciation. The exception is *D. noxia*, which is not found in eastern Asia (Blackman & Eastop 2006), and its distribution does not overlap with that of *A. certus*. Vouchers of the parasitoids and aphids are kept at −20°C in 100% molecular‐grade ethanol at the Beneficial Insect Introduction Research Unit, Newark, Delaware.

### 2.3 Measurement of parasitism

To measure parasitism, we exposed individual female parasitoids to the seven species of aphids listed above. We used females that were 1–5 days old and had been with males and aphids since emergence and thus had the opportunity to mate, host feed, and oviposit. To ensure that the females had a full egg load, we isolated females from aphids for 24 hr before using them in experiments (Wu & Heimpel, 2007). We put each female in a cage (10 cm diameter by 22 cm tall) enclosing the foliage of a potted plant of the appropriate species with 100 aphids of mixed instars of a single species. Female parasitoids were removed after 24 hr and were used only once. Ten days after exposure of aphids to parasitoids, we collected any mummified aphids and held them for adult parasitoid emergence. After the adults emerged, we recorded the number of mummified aphids and the number and sex of adult parasitoids.

Because *A. certus* females parasitized a mean of 6 aphids and a maximum of 37 aphids in 24 hr, the abundance of aphids and period of exposure allowed parasitoids to use their full egg complement. Furthermore, the density of aphids, amount of plant material, and cage size meant that parasitoids were unlikely to be limited by search rate. Therefore, we measured a combination of acceptance of hosts for oviposition and suitability of hosts for parasitoid development. However, other experiments have shown acceptance and mummification are positively correlated, except for aphids on toxic plants (e.g., *Asclepias*) or aphids protected by defensive endosymbionts (Desneux, Barta, Hoelmer, Hopper, & Heimpel, 2009; Hopper, Lanier, Rhoades, Hoelmer et al., 2017), which were not included in this study.

### 2.4 Analysis of parasitism, adult emergence, and progeny sex ratio

Because we were interested in the differences among parasitoid populations in their use of different aphid species, we used generalized linear models (GLM) to test the effects of parasitoid population and the interaction between aphid species and parasitoid population on the number of parasitized (mummified) aphids, adult emergence rates (proportion of parasitized aphids from which adult wasps emerged), and the proportion of males among adult parasitoid progeny. To examine the role of plant species, we used GLM to test differences in parasitism among aphid species on the same plant species (*H. vulgare*), and differences in parasitism among aphid species on different plant species (*Glycine max*, *Gossypium hirsutum*, and *Raphanus sativus*). The experimental unit for these analyses was a female parasitoid exposed to a single aphid species. Some dependent variables had non‐normal distributions with variances proportional to means, so we used the appropriate distributions (e.g., normal, negative binomial) for the dependent variables. We chose the distribution that gave highest model probability calculated from the residual deviance divided by residual degrees of freedom compared to a chi‐square distribution (Littell, Milliken, Stroup, & Wolfinger, 1996). The negative binomial distribution gave the best fit for the number of parasitized aphids, and the normal distribution gave the best fit for adult emergence rate and sex ratio. Aphid species and parasitoid population interacted in their effects each of the dependent variables, so we analyzed the differences among parasitoid populations in parasitism, adult emergence rates, and proportion males for each aphid species separately, using generalized linear models with the appropriate error distributions. We used Hochberg's adjustment to correct for multiple comparisons (Hochberg, 1988). For these analyses, we used the glm.nb function in the MASS R package (version 7.3‐48; Venables & Ripley, 2002) and the glm function in the stats package in R. We calculated least‐squares means and 95% asymptotic confidence intervals using the lsmeans function in the lsmeans R package (version 2.27‐61; Lenth, 2016). For some aphid species, the confidence intervals were sometimes asymmetrical; in these cases, we report means and asymptotic 95% confidence levels in the following format: mean [lower confidence level – upper confidence level].

Replicates in which females were not recovered (or died before the end of the exposure period) were not used, and after removing these replicates (7% of the experimental units), we had data for 6–14 females (mean of 10 females) from 16 parasitoid populations on each of the seven species of aphids for a total of 1,163 females. To reduce inadvertent laboratory selection, we tested the populations within three years of collection. We also used GLM to test the effects on parasitism of generations in culture and the interaction between aphid species and generations in culture. Because of space constraints, we were able to measure responses of a maximum of 120 females on the same date. To avoid confounding the effects of population differences with differences in dates tested, we spread the replicates for each population over multiple dates and tested several populations on each date. The seven populations collected in 2002–2003 were all tested together on five dates in 2004–2005. The three populations collected in 2004–2005 were all tested together on four dates in 2006. The two populations collected in 2006 were tested together on three dates in 2007. The remaining four populations, collected in 2001, 2007, 2008, 2010 (i.e., at the beginning and end of the project), were each tested on one or two dates. For analysis of adult emergence rate, we only used replicates where at least four aphids were mummified, and for analysis of sex ratio, we only used replicates where at least four adult parasitoids emerged, so the replicate numbers were lower for these variables.

Using Spearman's rho in the cor.test function in the stats package in R, we tested the correlations between adult emergence rates and (a) the proportion of male progeny and (b) the number of parasitized aphids. If females prefer aphid species on which their progeny are fittest, one would expect a correlation between these performance measures and parasitism rates, such that proportion males would be lowest and adult emergence rates would be highest for the aphid species with the highest parasitism.

### 2.5 Geographical distances and differences in parasitism

We tested the relationship between geographical distance and differences in parasitism among the Asian populations of *A. certus*. Because the population of *A. certus* in North America has only very recently established and thus is unlikely to have differentiated from Asian populations, we excluded it from this analysis. We calculated great‐circle distances between collection sites using latitudes and longitudes for each site (Table S1) and the distm function with the haversine method in the geosphere R package (version 1.5‐7; Hijmans, 2016). We calculated Mahalanobis distances among parasitism means for the aphid species using the dist.quant function in the ade4 R package (version 1.7‐10; Dray & Dufour, 2007). Because *Diuraphis noxia* was rarely parasitized by *A. certus* females from any population, we excluded it from these calculations. Using Mantel's permutation test, we compared the geographical and host use distance matrices with the mantel.randtest function in the ade4 R package with 10,000 permutations. We also graphed the similarity in parasitism among parasitoid populations, using the qgraph R package (version 1.4.4; Epskamp, Cramer, Waldorp, Schmittmann, & Borsboom, 2012)**. **In this network graph, the widths of the lines joining the populations are proportional to the similarity in parasitism, which was calculated as the maximum Mahalanobis distance for all populations minus the observed distance between each pair of populations. We also clustered the populations using the Mahalanobis distances in the hclust function in the base R package and tested whether clustering was associated with region (China–Inner Mongolia, China–Northeast, China–Hebei, Japan, Korea) using the adonis function in the vegan R package (version 2.4‐5; Oksanen et al., 2017), with 999 permutations.

### 2.6 Genome sequencing, assembly, and annotation

To determine orthology of SNP loci and find genes in *A. certus* that might be involved in differences in host use, we sequenced, assembled, and annotated the genome of one *A. certus* population (VJp01_TU). The sequencing library consisted of DNA from 24 male progeny from a single female extracted using the Qiagen DNeasy Blood and Tissue Kit (Qiagen, Valencia, CA). The DNA was used to make a genomic library with the Illumina TruSeq DNA LT Library Preparation Kit (Illumina, San Diego, CA), and paired‐end sequencing was conducted on an Illumina HiSeq 2500 at the Delaware Biotechnology Institute, Newark, Delaware.

Quality trimming and de novo assembly were done with the CLCBio Genome Workbench (version 5.0; http://www.clcbio.com) running on the bioinformatics cluster at the Delaware Biotechnology Institute, Newark, Delaware. We trimmed the Illumina sequences with a quality limit of 0.05 and an ambiguous limit of five nucleotides. After trimming, the read lengths averaged 144 nucleotides and the paired‐distances were 208–518 nucleotides. For assembly, we used a bubble size of 50, a word size of 25, and minimum contig length of 200 nucleotides.

For gene discovery, we used AUGUSTUS (version 3.3; Stanke & Morgenstern, 2005), trained with the *Nasonia* gene set. We searched for homologs of the identified amino acid sequences in the RefSeq database (accessed 4/21/2018; ncbi.nlm.nih.gov) using BLASTP (version 2.2.22; Altschul, Gish, Miller, Myers, & Lipman, 1990). Assembly completeness was assessed with BUSCO according to conserved arthropod gene content (version 3.0.2; Simão, Waterhouse, Ioannidis, Kriventseva, & Zdobnov, 2015).

### 2.7 Genetic distances and differences in parasitism

To generate data for phylogenetic and population genetic analyses, we used next‐generation sequencing of reduced‐representation genomic libraries to genotype single nucleotide polymorphisms (SNPs) among the 16 *A. certus* populations. Libraries were prepared as described in Manching et al. (2017). Briefly, genomic DNA was extracted from pools of wasps from each population using Qiagen DNeasy Blood and Tissue Kits (Qiagen, Valencia, CA), following the standard protocol. The resulting DNA was digested with restriction endonucleases using one rare cutter (*NgoMIV* with a 6 bp recognition site) and one frequent cutter (*CviQI* with a 4 bp recognition site) (New England Biolabs, Inc., Ipswich, MA), which together determined the number of unique locations of fragments across the genome and the lengths of these fragments. Custom adaptors, with barcodes for each population that also served to register clusters on the Illumina HiSeq during sequencing, were ligated onto the fragments using T4 ligase (New England Biolabs, Inc., Ipswich, MA). The ligates were pooled and purified using Agencourt AMPure XP beads (Beckman Coulter, Indianapolis, IN). The purified ligate was separated into 10 aliquots that were amplified in separate PCRs to both increase copy number at each locus and add more adaptor sequence for sequencing. The adaptors were designed so that the only fragments that amplify would have the rare‐common combination of cut sites. After PCR, the products were pooled and then size‐selected (300–350 bp) using the BluePippin system (Sage Science, Beverly, MA). After quantification with qPCR, the resulting fragments were sequenced for ~100 nucleotides in single‐end reads an Illumina HiSeq 2500 (Illumina, San Diego, CA) at the Delaware Biotechnology Institute.

Sequence data were processed with a reduced‐representation computational pipeline called RedRep (described in Manching et al. 2017; the scripts and documentation for the pipeline are available under an open source MIT license at https://github.com/UD-CBCB/RedRep. Briefly, sequences were deconvoluted by barcode using custom scripts and the FASTX‐Toolkit (version 0.0.14; http://hannonlab.cshl.edu/fastx_toolkit). Custom scripts and CutAdapt (version 1.14; Martin, 2011) were then used to remove adapters, trim low quality read ends, and filter out sequences that did not meet minimum length/quality standards or did not meet expectations for the restriction‐site sequences. High‐quality reads were mapped to the draft genome of *A. certus* using BWA‐MEM program (version 0.7.16a; Li, 2013). SNP loci were identified using the GATK HaplotypeCaller (version 3.5‐0; McKenna et al., 2010). We filtered the SNP loci for read depth ≥50 and then for presence in all populations using BEDtools (version 2.26) and custom scripts written in R (version 3.3.3; R.Core.Team, 2017). One population, VKor08_A, did not provide sufficient data for analysis of SNP frequencies.

We used SNP data to generate a parsimony‐based molecular phylogeny with the branch‐and‐bound algorithm in PAUP* (4.a build 159; Swofford, 2002) and estimated internal node support with the bootstrap algorithm in PAUP*. Because there was no bootstrap support for internal nodes, but strong genetic differentiation among populations, we tested the relationship between host use distance and genetic distance, as measured by *F*_ST_. Because *A. certus* individuals were pooled within populations to make the libraries for sequencing, we used read depths to estimate allele frequencies for SNP loci. We filtered the data for SNP loci that were present in all populations and had read depth ≥50, and we used the numbers of individuals in each pool in calculating *F*_ST_ between populations with the calcPopDiff function in the polysat R package (version 1.7‐2; Clark, 2017). Using Mantel's permutation test, we compared the genetic and parasitism distance matrices (10,000 permutations with the mantel.randtest function in the ade4 R package).

To explore the relationship between genetic differentiation and differences in parasitism, we determined whether the position of reduced‐representation loci in the genome was genic (defined as occurring either within a gene or ≤10 kb upstream or downstream of a gene) or nongenic and then categorized genic loci based on SNP content (defined as “with SNP” or “without SNP”). We compared the functions of genes near reduced‐representation loci with versus without SNP loci. To determine the function of these genes, we compared their amino acid sequences to those of proteins in the RefSeq database (accessed 4/21/2018; ncbi.nlm.nih.gov) using BLASTP, and we conducted domain analyses with InterProScan (version 5; Jones et al., 2014).

## 3 RESULTS

### 3.1 Differences in parasitism among parasitoid populations and aphid species

**Figure 1:**
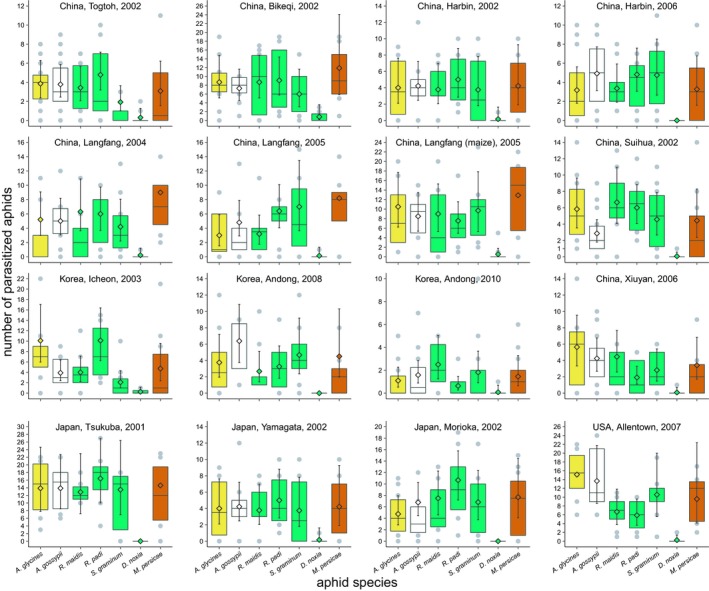
Host specificity of populations of *Aphelinus certus* in laboratory experiments. Parasitoid populations are indicated by city, region/country, and year of collection. Diamonds are means and vertical lines are asymptotic 95% confidence intervals. Tops and bottoms of the boxes indicate lower and upper quartiles, and the middle horizontal lines indicate medians. Gray dots are outliers beyond the quartiles. Host plants on which aphids were exposed are indicated by the fill colors of the boxes: green = barley; brown = radish; yellow = soybean; white = cotton

Parasitoid population and the interaction between aphid species and parasitoid population strongly affected parasitism (Figure 1; GLM for number aphid parasitized [negative binomial] = parasitoid population +aphid species × parasitoid population; parasitoid‐population model deviance = 308; residual deviance = 1,898; *df* = 15, 1,147; *p* < 0.00001; interaction model deviance = 642; residual deviance = 1,256; *df* = 96, 1,051; *p* < 0.00001). Parasitism differed among populations for all aphid species except *D. noxia*, which was rarely parasitized by any parasitoid population (Figure 1, Table 1). Parasitism of *Aphis glycines* varied 14‐fold among populations, despite all but one of them having originally been collected from *A. glycines*. Since entering culture, all 16 parasitoid populations were reared on *A. glycines* for various numbers of generations (6–44) before parasitism was measured. However, neither generations in culture nor the interaction between aphid species and generations in culture affected parasitism (GLM for number aphid parasitized [negative binomial] = aphid species + generations in culture + aphid species × generations in culture; aphid species model deviance = 434; residual deviance = 1,256; *df* = 6, 1,156; *p* < 0.00001; generations‐in‐culture model deviance = 0.8; residual deviance = 1,255; *df* = 1, 1,155; *p* = 0.36; interaction model deviance = 3; residual deviance = 1,252; *df* = 6, 1,149; *p* = 0.86). Nor was *A. glycines* among the most favored aphid species: Only two populations parasitized *A. glycines* more than other aphid species, while *M. persicae* and *R. padi* were the most parasitized aphid species in four and five populations, respectively. Excluding *D. noxia*, the aphid species with maximum and minimum parasitism varied among parasitoid populations, with five of the six aphid species having maximum and minimum parasitism by females from at least one parasitoid population. Parasitism varied among the aphid species on *H. vulgare* (GLM for number aphid parasitized [negative binomial] = aphid species; model deviance = 421; residual deviance = 660; *df* = 3, 648; *p* < 0.00001), from little or no parasitism of *D. noxia* by any population to maximum parasitism of *R. padi* by five populations (Figure 1). On the other hand, parasitism did not differ among aphid species on plant species (*Glycine max*, *Gossypium hirsutum*, and *Raphanus sativus*) with very different chemistries (GLM for number aphid parasitized [negative binomial] = plant species; model deviance = 1; residual deviance = 594; *df* = 2, 508; *p* = 0.78).

**Table 1:** Analyses of deviance for differences among *Aphelinus certus* populations in numbers aphids parasitized, adult parasitoid emergence rates, and progeny sex ratios on each aphid species

Variable	Aphid species	Model		Residual		Raw *P*	Hochberg *P*
		*df*	Deviance	*df*	Deviance		
Numbers of aphids parasitized	*Aphis glycines*	15	70.3	149	193.0	<0.00001	0.02^a^
	*Aphis gossypii*	15	74.9	160	207.8	<0.00001	0.03^a^
	*Rhopalosiphum maidis*	15	42.9	148	185.8	0.0002	0.01^a^
	*Rhopalosiphum padi*	15	101.1	150	189.9	<0.00001	0.05^a^
	*Schizaphis graminum*	15	48.6	152	196.1	<0.00001	0.01^a^
	*Myzus persicae*	15	41.1	154	198.2	0.0003	0.01^a^
	*Diuraphis noxia*	15	22.7	138	56.6	0.09	0.01
Adult emergence rates	*Aphis glycines*	15	0.6	76	0.9	<0.00001	0.05^a^
	*Aphis gossypii*	15	0.2	87	1.8	0.72	0.01
	*Rhopalosiphum maidis*	15	0.4	73	1.1	0.04	0.01
	*Rhopalosiphum padi*	14	0.7	89	1.5	0.00005	0.03^a^
	*Schizaphis graminum*	15	0.3	68	0.6	0.01	0.02^a^
	*Myzus persicae*	15	1.0	78	2.7	0.02	0.01
Proportion males among progeny	*Aphis glycines*	14	1.5	66	3.5	0.01	0.05^a^
	*Aphis gossypii*	15	0.8	75	3.5	0.27	0.01
	*Rhopalosiphum maidis*	15	1.1	64	3.9	0.23	0.01
	*Rhopalosiphum padi*	15	1.1	64	4.9	0.46	0.01
	*Schizaphis graminum*	14	1.4	77	4.2	0.04	0.03
	*Myzus persicae*	15	1.0	65	3.1	0.17	0.02

a Raw *P* ≤ Hochberg *P;* experiment‐wise error rate α = 0.05.

**Figure 2:**
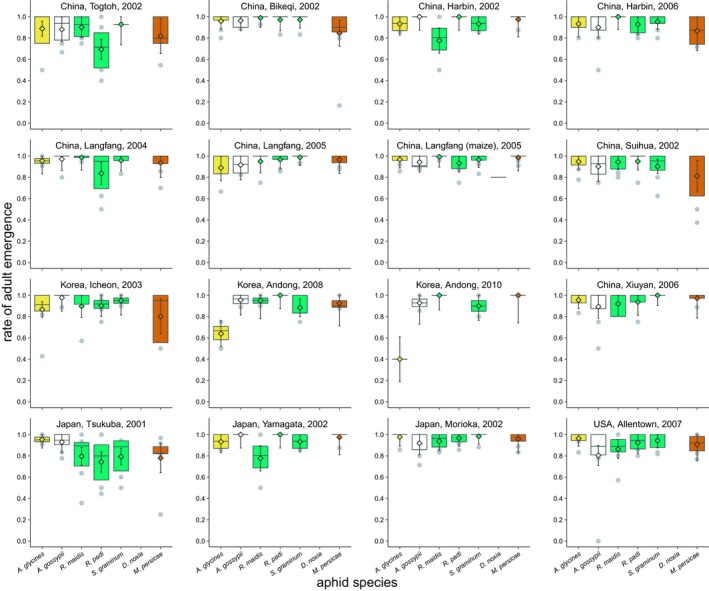
Adult emergences from seven species of aphids parasitized by *Aphelinus certus*. See Figure 1 for key to symbols and colors

Parasitoid population and the interaction between aphid species and parasitoid population affected the emergence rates of adult progeny of *A. certus* (Figure 2, GLM for number of adults per aphid parasitized [normal] = parasitoid population +aphid species x parasitoid population; parasitoid‐population model deviance = 1; residual deviance = 11; *df* = 15, 551; *p* < 0.00001; interaction model deviance = 2; residual deviance = 9; *df* = 80, 471; *p* < 0.00001). However, more than 90 percent of the combinations of aphid species and parasitoid populations showed mean emergence rates ≥0.80. Differences in emergence rates were only found for three aphid species (Table 1): Emergence from *A. glycines* ranged from a low of 0.40 [0.19–0.61] to a high of 0.98 [0.89–1.00], emergence from *R. padi* ranged from a low of 0.69 [0.60–0.79] to a high of 0.97 [0.89–1.00], and emergence from *S. graminum* ranged from a low of 0.79 [0.72–0.87] to a high of 0.99 [0.91–1.00]. Emergence rate decreased with the number of aphids parasitized, although the slope was shallow and the correlation weak (Spearman's ρ = −0.10; *df* = 1,163; *p* = 0.02).

**Figure 3:**
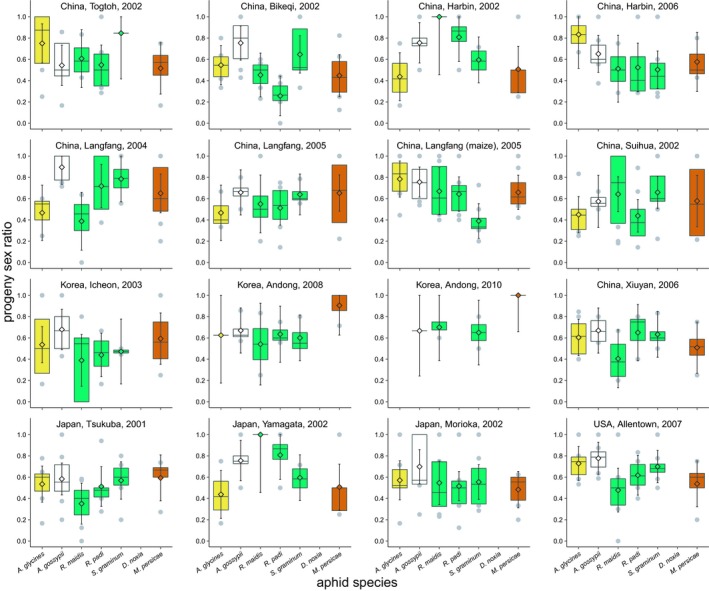
Proportion of males among adult progeny emerging from seven species of aphids parasitized by *Aphelinus certus*. See Figure 1 for key to symbols and colors

Parasitoid population and the interaction between aphid species and parasitoid population affected the proportion of males among *A. certus* progeny (parasitoid population +aphid species x parasitoid population; parasitoid‐population model deviance = 2; residual deviance = 29; *df* = 15, 489; *p* = 0.0003; interaction model deviance = 6; residual deviance = 23; *df* = 78, 411; *p* = 0.01), but only one aphid species, *A. glycines*, showed significant variation in proportion of males (Table 1). Proportion males from *A. glycines* ranged from a low of 0.34 [0.17–0.51] to a high of 0.83 [0.52–1.00]. Nonetheless, proportions were male‐biased (lower 95% confidence interval>0.50) for two to six *A. certus* populations depending on the aphid species, and female‐biased (lower 95% confidence interval <0.50) for one *A. certus *population on *R. padi*, with *R. maidis* being the only aphid species with no biases (Figure 3). The proportion of males decreased with the numbers of aphids parasitized (Spearman's ρ = −0.14; *df* = 102; *p* = 0.002), indicating that female *A. certus* laid more fertilized eggs in aphids that they parasitized more.

### 3.2 Geographical distances and differences in parasitism

**Figure 4:**
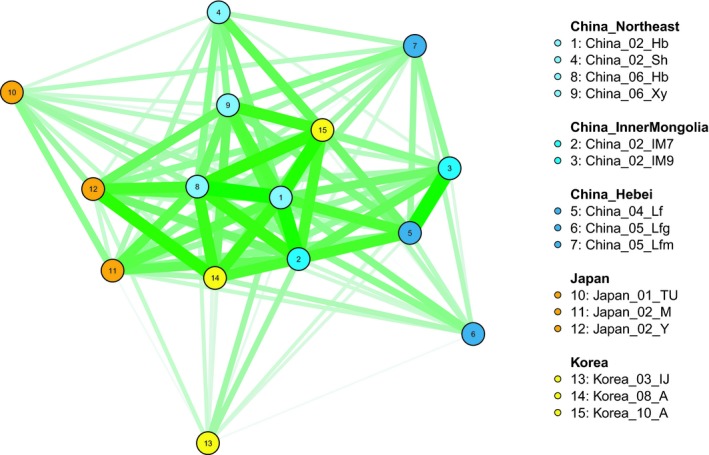
Network graph of the similarity in parasitism among parasitoid populations**. **The widths of the lines joining the populations are proportion to parasitism similarity, which was calculated as the maximum Mahalanobis distance for all populations minus the observed distance between each pair of populations

Geographical distances between the Asian *A. certus* populations ranged from 35 km to 2,574 km. The closest populations were the two from Inner Mongolia, and the most distant were those from Inner Mongolia and Japan. A network graph of the similarity in parasitism among *A. certus* populations suggested some geographical partitioning (Figure 4), but populations from the same region did not cluster significantly together (ANOVA for Mahalanobis distance = region; *F* = 1.3; *df* = 4, 9; *p* = 0.15). Furthermore, differences in parasitism among populations did not correlate with geographical distance between populations (Mantel's test; Pearson's *r = *0.14; *p* = 0.16).

### 3.3 Genome sequencing, assembly, and annotation

With 30 Gb of paired‐end Illumina data and 103x coverage, our assembly of the *A. certus* genome was 290 Mb long, which is 20% smaller than the 361 Mb genome size estimated from flow cytometry (Gokhman, Kuhn, Woolley, & Hopper, 2017). The assembly had an N_50_ of 15 Kb and had 34 k contigs with lengths ≥1 Kb. The difference in estimates of sizes between assembly and flow cytometry, as well as the fragmented assembly, may result from repetitive DNA content, which is difficult to assemble. Nonetheless, the assembly captured a fairly complete gene set, as measured by BUSCO (Simão et al., 2015), having 92% of the core arthropod genes. Using AUGUSTUS, we found 27,315 genes, which together comprise 26 Mb of DNA sequence or 9% of the genome assembly. With BLASTP (*E ≤ *0.001), we found homologs for 78% (19,791) of these genes in the RefSeq database.

### 3.4 Genetic distances and differences in parasitism

Sequencing the reduced‐representation libraries from these *A. certus* populations and mapping the sequences onto our draft assembly of the *A. certus* genome yielded 18 K‐163 K reduced‐representation loci, depending on the population (Table S2). The exception was the library for the population from Korea in 2008, which had much lower coverage and fewer loci and was therefore excluded from further analysis. Among the reduced‐representation loci, 1,707–7,636 had ≥50× coverage (medians of 145–324 reads per locus), depending on the population, and 780 reduced‐representation loci were shared by all populations. We used this set of 780 loci for all subsequent analyses and found that 371 of them harbored 892 SNPs (range 1–8 SNPs per locus; 228 loci [61%] had 1 SNP). The median locus length was 94 nucleotides, so there were ~1.2 SNP per 100 nucleotides (892/780 x 100/94). The combined length of these loci is 73 Kb, which is ~0.02 percent of the genome length. We used this set of 892 SNP loci for phylogenetic and population genetic analyses.

Only 36 SNP loci were parsimony‐informative, and the resulting phylogeny had bootstrap support values <30% for all internal nodes. All of the genetic differences were among populations at the tips of the phylogeny, so rather than mapping host use onto the molecular phylogeny, we analyzed the genetic distances among populations and tested the correlation between differences in parasitism and genetic distances.

**Figure 5:**
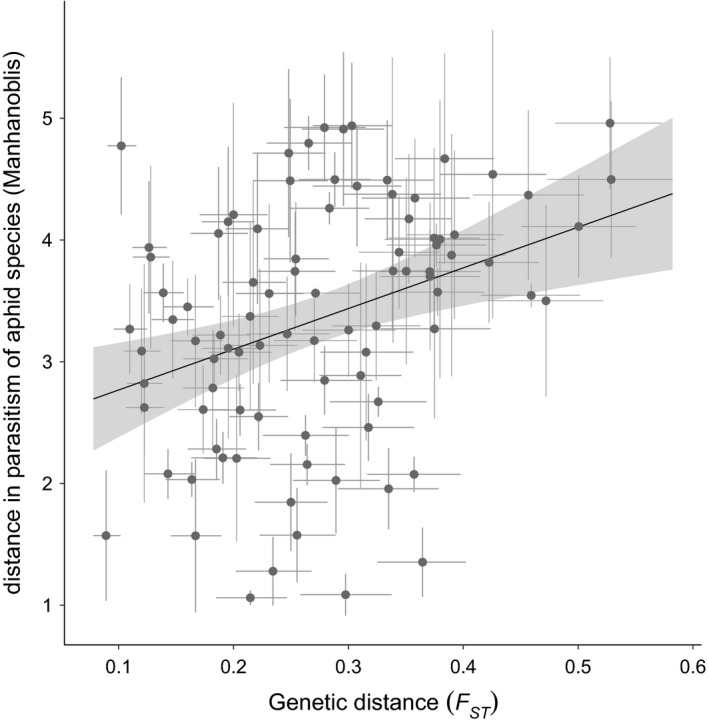
Relationship between differences in parasitism of aphid species and genetic distances (*F*_ST_) among 15 populations of *Aphelinus certus*. Points are observations, error bars are 95% bootstrap confidence intervals (*n* = 10,000), the sloped line is a linear regression, and the gray area is the 95% confidence interval for the regression

Between‐population genetic distances, as measured by *F*_ST_ averaged across 892 SNP loci, ranged from 0.09 to 0.53 (Figure 5), with the lowest value being between the populations from Andong, South Korea, and Xiyuan, China, and the highest value being between populations from Icheon, South Korea, and Suihua, China. With the exception of nine values between 0.09 and 0.15, the remaining 96 values for *F*_ST_ were in the ranges that Hartl and Clark (1997) consider to indicate great (0.15–0.25) or very great (>0.25) genetic differentiation. The minimum and maximum *F*_ST_ for each population ranged from 0.09–0.31 for Andong, South Korea, to 0.30–0.53 for Suihua, China. The differences in parasitism of aphid species correlated with *F*_ST_ (Mantel's test; Pearson's *r = *0.38; *p* = 0.04; Figure 5), and genetic divergence explained 14 percent of the variation in the differences in parasitism.

**Figure 6:**
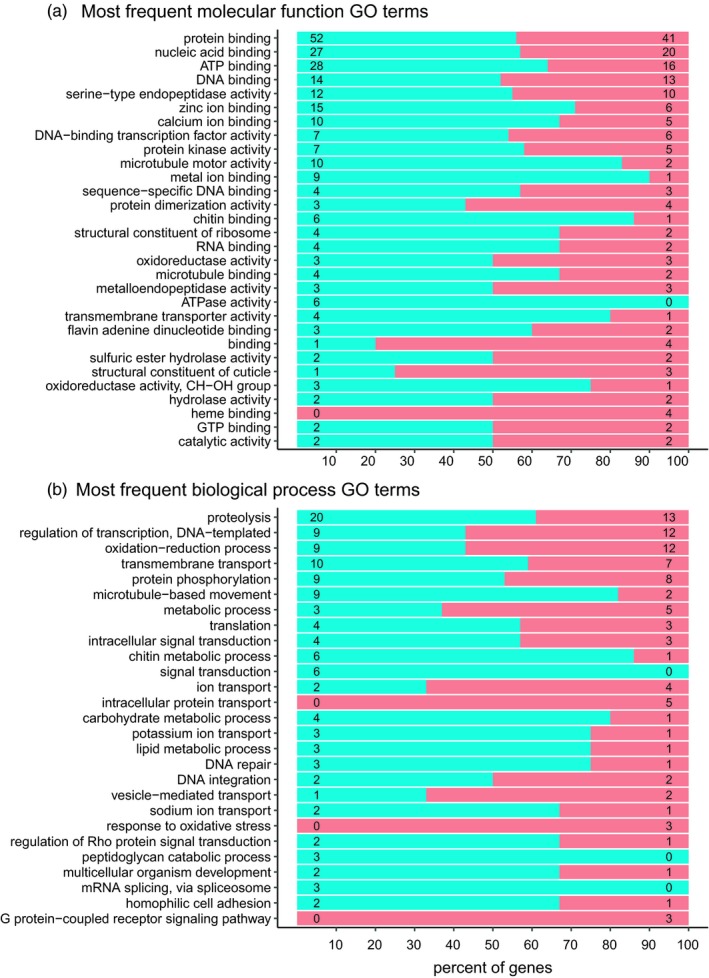
Frequencies of genes with different functions near reduced‐representation loci with SNP 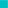 versus without SNP 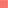 for various gene ontology annotations of (a) molecular function and (b) biological process. One hundred percent stacked graph is shown; numbers on the bars are the frequencies of genes with the indicated annotation.

Because we found an association between genetic divergence and parasitism, we went on to analyze whether there was an association between the presence of polymorphisms and differences in gene function. The reduced‐representation loci and the SNP loci they harbored were widely distributed across the *A. certus* genome (Fig. S1). For the 780 reduced‐representation loci present in all populations with ≥50× coverage, 260 mapped to contigs that did not contain genes. The remaining 520 loci (67%) mapped to contigs that contained a total of 1,359 genes. When we examined the locations of loci in these contigs, we found that 90% were in or near (within 10 Kb) 760 genes (the remaining genes on these contigs were at least 10 kb away from any reduced‐representation locus). Since not all of the reduced‐representation loci had SNPs, we examined their position relative to their SNP status. We found 432 genes near loci with SNPs, and 328 genes near loci without SNPs. Among the with‐SNP genes, 405 had BLASTP hits in the RefSeq database, and 302 had gene ontology (GO) annotations. Among the without‐SNP genes, 306 had BLASTP hits in the RefSeq database, and 213 had GO annotations. Several molecular functions and biological processes were present only among the with‐SNP (e.g., mRNA splicing via spliceosome) or without‐SNP genes (e.g., G protein‐coupled receptor signaling pathway) (Figure 6), but none of these differences were significant in enrichment tests when corrected for multiple comparisons (Fisher's exact test, False Discovery Rate = 0.05).

## 4 DISCUSSION

Although the 16 *A. certus* populations we studied do not differ in the range of aphid species they parasitize, they have diverged in their levels of parasitism of species within that range. Adaptation to local differences in abundance of aphid species might cause such divergence (Forbes et al., 2009; Hopper, De Farias, Woolley, Heraty, & Britch, 2005). The lack of a relationship between geographical distance and host use patterns, together with the similarity in host use patterns of populations from different regions, suggests that patterns of host specificity are mosaic rather than clinal. The correlation between genetic differentiation and differences in host use indicates that genetic differentiation could explain 14% of variation in host use, but the remaining genetic variation may have nothing to do with differences in host use. It might be driven by other adaptations, drift, or mutation. However, that we found any relationship between genetic differences and differences in host use is surprising, given our limited sampling of the *A. certus* genome.

Our results imply that adaptation to different aphid species is quite local, that is at the level of counties rather than countries, because populations separated by 35–103 km showed differences in parasitism of aphid species. Variation in aphid abundances may reflect the spatial scale of crop and noncrop patchiness in the regions where these populations were collected. Unfortunately, we lack historical data for variation in abundances of these aphids. Nonetheless, several other lines of evidence suggest that adaptation to locally abundant aphids is likely for these parasitoid populations. First, dispersal rates for species in the genus *Aphelinus* are quite low, on the scale of meters per generation (Fauvergue & Hopper, 2009), with searches for mates and hosts primarily while walking (De Farias & Hopper, 1997; Fauvergue et al., 1995). Although *A. certus* does parasitize adult aphids, it rarely parasitizes winged adults, so phoresy in parasitized adult aphids is unlikely to increase parasitoid dispersal distances. Second, the lack of a positive relationship between adult emergence and the level of parasitism suggests that *A. certus* females may be adapted to use whichever aphid species is most abundant locally. Thus, rarely encountered aphid species are not judged suitable, even when progeny fitness would be high in them. Third, *D. noxia* is rarely parasitized by any of these *A. certus* populations, perhaps because of their nonoverlapping distributions. The geographical range of *D. noxia* lies well outside that of *A. certus*, with the nearest *D. noxia* populations in far western China, and even there, *D. noxia* is a recent invader (Zhang, 1991). In behavioral observations, *A. certus* females ignore *D. noxia* when encountered, so that low parasitism appears to come from low acceptance for oviposition, rather than low suitability as a host. This may arise from loss of chemoreceptors for recognizing *D. noxia* as a host. This is not the case for the other aphid species, but for these, we could not distinguish differences in whether eggs were laid or progeny failed to develop to aphid mummification.

Our results match the local adaptation found in one of the few other studies of geographical variation in host specificity, where parasitoids have adapted to the songs of locally abundant cricket species (Gray et al., 2007). The results so far on local adaptation to host species in parasitoids resemble findings about adaptation to host plant species in herbivores (Via & Hawthorne, 2002) or to microclimate in *Drosophila* (Nevo, Rashkovetsky, Pavlicek, & Korol, 1998) rather than the results about parasitoids on different populations within host species (Dupas et al., 2003; Kraaijeveld & Godfray, 2001).

A caveat to our speculations about local adaptation is that the differences we observed in patterns of host use among *A. certus* populations might change if we used the aphid populations from their collection sites. In particular, aphid populations may vary in the presence of secondary endosymbionts that can protect aphids from parasitism by some parasitoids (Oliver, Degnan, Hunter, & Moran, 2009; Oliver, Russell, Moran, & Hunter, 2003). However, the *Aphelinus* species we have tested are not affected by *Hamiltonella defensa* in *Aphis craccivora* (Hopper et al., 2018). Furthermore, research in some other systems has shown little evidence of local adaptation by parasitoids to different populations of the same host species (Dupas et al., 2003; Kraaijeveld & Godfray, 2001). This is so despite evidence for genetic variation in parasitoids for ability to overcome resistance in hosts (Rolff & Kraaijeveld, 2001), although in some cases this variation may involve cryptic species of parasitoids that are separated by their own endosymbionts (Vorburger, Sandrock, Gouskov, Castaneda, & Ferrari, 2009). Indeed, the geographical variation in foraging behavior of *Leptopilina clavipes* for *Drosophila* species on different substrates probably involves incipient or cryptic species (Pannebakker et al., 2008).

Host plant species did not affect which aphid species were parasitized by these parasitoid populations. The differences in parasitism among aphid species on *H. vulgare* and similarity in parasitism of aphid species on host plants with very different chemistry both suggest that host plant was unimportant in determining which aphid species were parasitized. This is not to say that host plant is never important for aphid parasitoids. For example, *Bionodoxys communis* produces fewer mummies on *Aphis nerii* and *Aphis asclepiadis* when the aphids are on plants with higher levels of toxins (Desneux et al., 2009), and *Aphelinus* species show a similar reduction in mummy production when exposed to *Aphis gossypii* on *Asclepias syriaca* which produces cardenolides versus *G. hirsutum* which produces none (K. R. Hopper, unpublished data).

Given that we argue here that these parasitoid populations have adapted to locally abundant or high‐quality aphids in the field, one might expect that 6–44 generations of laboratory rearing on *Aphis glycines* would select for greater use of *Aphis glycines*. However, parasitism was not affected by generations in culture or the interaction between aphid species and generations in culture. Henry et al. (2008) found a strong response to selection in *Aphidius ervi* for parasitizing a novel host, and adaptation to the novel host reduced fitness on its original host. However, that experiment involved adaptation to an initially very poor host and the response to selection was measured 50 generations after selection started. The lack of change in patterns of parasitism with generations in culture that we observed may have resulted from several factors: low initial genetic variation in ability to parasitize the various aphid species, low population sizes (about 800 adult parasitoids per generation), and few generations in culture before testing. Our rearing procedures were designed to maintain existing genetic variation (Hopper et al., 1993), but existing variation may be low in a locally adapted population. If genetic variation is lacking initially, its creation requires mutations that are likely in field populations (which number in the millions or billions and reproduce for hundreds to thousands of generations), but extremely unlikely in our small laboratory populations reared for a few generations on *Aphis glycines*.

Low dispersal rates and high levels of genetic differentiation suggest that gene flow is restricted among these populations of *A. certus*, and this increases the likelihood of their adaptation to local differences in abundances or quality of aphid species. Indeed, we found that genetic divergence, as measured by *F*_ST_, explained 14 percent of the variance in parasitism of aphid species. The estimates of genetic divergence came from SNP loci in reduced‐representation loci that comprised ~0.02 percent of the genome and were within 10 Kb of 760 genes that comprised ~3 percent the genes in the genome. Given this limited sample of the genome and genes of *A. certus*, two hypotheses might explain why we found a relationship between genetic divergence and differences in parasitism: (a) Many genes across the genome are involved, each having small effects, and our limited sample captured enough of them to explain 14 percent of the variance in parasitism—in this case, any randomly selected set of ~800 genes would explain a similar amount of variation; or (b) a few genes are involved, each with large effects, and our limited sample fortuitously hit a few of these genes, which together explained 14 percent of the variation in parasitism—in this case, a different set of genes might well explain zero percent of the variance. With the available information, we cannot distinguish between these hypotheses. However, we had hoped that analysis of differences in the functions of genes with versus without SNP loci near them might reveal candidates for local adaptation in parasitism. However, we found no enrichment or depletion of the functions of the proteins coded by the two sets of genes.

## 5 CONCLUSIONS

Parasitic wasps are among the most species‐rich groups on Earth, and a major cause of this diversity may be adaptation to local variation in the presence or abundance of host species. Little is known about local patterns of parasitoid host use, and the research presented here is the largest such study conducted to date. Our research on geographical variation in host use among 16 populations of an aphid parasitoid, *Aphelinus certus*, showed that although the parasitoid populations did not differ in host range (i.e., the set of species they will parasitize), they did differ in levels of parasitism of the aphid species tested. Interpopulation differences in parasitism could not be explained by the geographical distance between populations, nor did clustering of populations by parasitism patterns correlate with geographical regions. Instead, we found that interpopulation differences in parasitism were associated with patterns of genetic differentiation, as measured by *F*_ST_ for SNPs distributed across the genome. Of the 760 genes we examined, those with SNPs did not differ in function from those without SNPs. This suggests that interpopulation variation in the sequence or expression level of genes with the same basic functions may be responsible for local differences in host specificity, which stands in contrast to our initial expectation that genes in a limited set of functional categories would be involved in local adaptation. In any case, this is one of the few studies of local differentiation in patterns of parasitism that provides evidence that local adaptation has an underlying genetic basis. Future studies will be needed to determine whether such striking levels of association between variation in host specificity and genetic differentiation are common to other insects that display local variation in host specificity.

## DATA ARCHIVING

Data from the host specificity experiments along with the *F*_ST_ estimates are archived on Ag Data Commons (DOI: dx.doi.org/10.15482/USDA.ADC/1503305). DNA sequence data are archived in the NCBI Sequence Read Archive (www.ncbi.nlm.nih.gov/sra; Accession Numbers SRR8307154‐SRR8307169 and SRR8307569).

## CONFLICT OF INTEREST

The authors declare no conflict of interest related to this manuscript.
