# Supplementary material for: Counties not countries: Variation in host specificity among populations of an aphid parasitoid
Source: Evol Appl. 2019 Jan 31;12(4):815–29. doi: 10.1111/eva.12759 (PMC6439487; doi:10.1111/eva.12759)
Supplement: Supplementary file 1 [file EVA-12-815-s001.docx]

**Fig. S1**. Distribution of *F_S_*_T_ values and contig lengths across the genome of *Aphelinus certus*. See text for details of library preparation, sequencing, and analysis of population divergence. The regions where the contig length doesn’t change indicate contigs with several SNP loci or reduced-representation loci (~400 of the latter did not harbor SNP).
